# Diagnostic Performance of Triglyceride/High-Density Lipoprotein Cholesterol (TG/HDL-C) Ratio in Detecting Insulin Resistance Among Obese Children and Adolescents

**DOI:** 10.7759/cureus.103072

**Published:** 2026-02-05

**Authors:** Semra Bahar Akin

**Affiliations:** 1 Pediatric Endocrinology, Derince Training and Research Hospital, Kocaeli, TUR

**Keywords:** adolescent, child, high-density lipoprotein cholesterol, insulin resistance, obesity, triglycerides

## Abstract

Background

We evaluated the diagnostic performance of the triglyceride/high-density lipoprotein cholesterol (TG/HDL-C) ratio for detecting insulin resistance (IR) in obese children and adolescents using sex- and puberty-specific homeostasis model assessment for insulin resistance (HOMA-IR) cutoffs.

Methods

This retrospective cross-sectional study included 207 obese patients (5-18 years old; 127 girls and 80 boys) assessed at a tertiary pediatric endocrinology clinic. IR was defined using sex-specific and pubertal stage-specific HOMA-IR thresholds. Anthropometric data and fasting glucose, insulin, lipid profile, glycated hemoglobin (HbA1c), and thyroid-stimulating hormone (TSH) were recorded. Receiver operating characteristic (ROC) analysis evaluated the TG/HDL-C ratio for IR discrimination.

Results

Compared with the IR-negative group, IR-positive participants had higher weight, body mass index (BMI) (and standard deviation score {SDS}), TG/HDL-C ratio, fasting insulin, HOMA-IR, HbA1c, fasting glucose, triglycerides, and TSH (all p<0.05). The best threshold for TG/HDL-C to identify IR was around 1.7 (area under the curve {AUC}=0.601; p=0.019), with sensitivity and specificity of 71.2% and 55.1%, respectively. Diagnostic accuracy was consistent across sex and pubertal groups.

Conclusions

The TG/HDL-C ratio demonstrates moderate diagnostic performance in detecting insulin resistance in obese children and adolescents, and this performance is independent of pubertal status or sex. In clinical settings, such as primary care, where insulin measurement and HOMA-IR calculation are not feasible, the TG/HDL-C ratio can be used as an independent marker. However, when insulin measurements are possible, this ratio should be considered a supportive marker alongside HOMA-IR. Additional investigations should assess the potential of this ratio to enhance diagnostic accuracy in multivariate analysis.

## Introduction

By 2022, the worldwide rate of obesity in individuals aged 5-19 had risen to 20%, corresponding to an estimated 390 million affected individuals, compared with 8% in 1990 [1]. This alarming rise has led to a significant increase in the burden of early metabolic complications during childhood and adolescence, making the accurate and timely identification of insulin resistance (IR) increasingly important in clinical practice. National surveys conducted in Turkey also indicate a steady increase in the prevalence of overweight and obesity during childhood [2,3].

Childhood obesity plays a significant role in the onset of various metabolic complications, including abnormal lipid profiles, reduced insulin sensitivity, and an increased risk of developing type 2 diabetes [4]. Therefore, insulin resistance and lipid disorders are frequently observed together in obese children and adolescents, increasing their long-term cardiometabolic risk [5]. The homeostasis model assessment for insulin resistance (HOMA-IR) is regarded as a more reliable indicator of IR than the fasting glucose-insulin ratio [6] and is therefore widely used in pediatric clinical and epidemiological studies [7]. However, because HOMA-IR is based on fasting insulin measurements, variability in insulin assay methods and limited routine availability across clinical settings restrict its use, particularly in primary healthcare and large-scale screening programs. Accordingly, alternative biochemical markers that indirectly reflect insulin resistance, are readily accessible, and are more practical for clinical application may be advantageous.

Recently, the triglyceride (TG)-to-high-density lipoprotein cholesterol (HDL-C) ratio has emerged as an indirect indicator of insulin resistance, particularly in obese pediatric populations [8]. Various studies have established a consistent association between the TG/HDL-C ratio and insulin resistance, suggesting that when evaluated alongside established indices such as HOMA-IR, this ratio may serve as a complementary marker for metabolic risk assessment [9]. However, marked variability in the reported cutoff values for the TG/HDL-C ratio across different populations indicates that its diagnostic utility may be influenced by demographic characteristics, pubertal status, lifestyle factors, and ethnicity [8-13]. In our country, studies examining the association between the TG/HDL-C ratio and IR in pediatric and adolescent populations, particularly among obese individuals, while accounting for sex- and puberty-specific HOMA-IR thresholds, remain limited. Consequently, it remains unclear to what extent the discriminatory capacity of the TG/HDL-C ratio is preserved when physiologically appropriate, sex- and puberty-adjusted HOMA-IR cutoff values are applied in clinical practice.

Therefore, we aimed to evaluate the diagnostic performance of the TG/HDL-C ratio as a screening/supportive marker for insulin resistance in obese children and adolescents using sex-specific and pubertal stage-specific HOMA-IR thresholds and to explore its potential utility in routine clinical settings.

## Materials and methods

Participants and study design

This retrospective, cross-sectional study was based on clinical data extracted from patient records of a tertiary care pediatric endocrinology outpatient service. According to the World Health Organization (WHO) growth standards, individuals whose body mass index (BMI) exceeded the age- and sex-specific mean by two or more standard deviations were categorized as obese [14]. Patients with syndromic obesity, chronic systemic disease, thyroid dysfunction, Cushing’s syndrome, or known genetic or metabolic disorders or those taking medications such as corticosteroids, antipsychotics, or drugs that could affect insulin sensitivity and lipid metabolism were excluded. Additionally, individuals with incomplete data or those with acute illness during the assessment were excluded from the analysis.

Assessment of pubertal status and anthropometric measurements

Pubertal status was determined by Tanner staging; boys with testicular volume of ≥4 mL and girls at Tanner breast stage of ≥2 were considered pubertal, while others were classified as prepubertal [15].

Standardized anthropometric procedures were applied, with body weight measured by an electronic scale (seca 703®, seca GmbH & Co. KG., Hamburg, Germany) and height obtained via a fixed stadiometer (seca 220®). BMI was calculated by dividing body weight (kg) by the square of height (m). BMI-standard deviation score (SDS) values were determined according to the WHO references.

Clinical and laboratory data

The demographic and clinical data of the participants included age, sex, anthropometric measurements (height, weight, BMI, and BMI-SDS), pubertal status, the presence of acanthosis nigricans, and blood pressure measurements. Blood pressure was classified according to the 2017 American Academy of Pediatrics (AAP) guidelines [16].

Laboratory data were obtained retrospectively from medical records and included fasting glucose, insulin, glycated hemoglobin (HbA1c), lipid profile (triglycerides {TG}, total cholesterol, low-density lipoprotein cholesterol {LDL-C}, and high-density lipoprotein cholesterol {HDL-C}), thyroid-stimulating hormone (TSH), free thyroxine (fT4), aspartate aminotransferase (AST), alanine aminotransferase (ALT), cortisol, and adrenocorticotropic hormone (ACTH).

Serum insulin, TSH, free T4, 17-hydroxyprogesterone, anti-thyroid peroxidase (POD) antibody, and anti-thyroglobulin antibody levels were measured using a chemiluminescent immunoassay (CLIA) method on the ARCHITECT i8000 analyzer (Abbott Diagnostics, Abbott Park, IL).

Plasma glucose was measured using the glucose oxidase-peroxidase (GOD-POD) method, and triglycerides were measured using the glycerol kinase-peroxidase method on the Mindray BS-2000 analyzer (Mindray, Shenzhen, China).

HDL-C and LDL-C levels were determined by direct enzymatic colorimetric assays, and total cholesterol was measured using the cholesterol oxidase-peroxidase (CHOD-POD) method.

AST and ALT levels were analyzed using UV kinetic methods without pyridoxal phosphate supplementation.

All biochemical analyses, including insulin measurements, were performed in the same central laboratory using the same analyzer and assay methodology throughout the study period.

Definition of insulin resistance

Insulin resistance was determined using HOMA-IR threshold values defined specifically for the sex and pubertal stages of children and adolescents in our country [7]. Insulin resistance was operationally defined according to HOMA-IR thresholds stratified by both sex and pubertal status. Among prepubertal participants, HOMA-IR values at or above 2.67 in girls and 2.22 in boys were accepted as indicative of insulin resistance. For pubertal individuals, higher cut-off points were applied, with insulin resistance defined by HOMA-IR values of ≥3.82 in girls and ≥5.22 in boys.

The HOMA-IR value was calculated by dividing the product of fasting glucose (mg/dL) and fasting insulin (µU/mL) levels by 405 [7,17]. The TG/HDL-C ratio was calculated using the fasting TG and HDL-C values.

Statistical analysis

The statistical evaluation of the dataset was undertaken using IBM SPSS Statistics version 29.0 (IBM Corp., Armonk, NY), together with MedCalc statistical software version 22.026 (MedCalc Software Ltd., Ostend, Belgium). The assessment of data distribution was based on the Shapiro-Wilk test. Variables with a normal distribution are reported as mean±standard deviation, while those not normally distributed are shown as median and interquartile range. Groupwise comparisons were performed with parametric or nonparametric tests according to data distribution, and categorical variables were analyzed using chi-square or Fisher’s exact tests.

Associations between HOMA-IR, the TG/HDL-C ratio, and additional clinical variables were explored through correlation-based analyses. Correlation coefficients were derived using Pearson’s method for normally distributed data and Spearman’s rank method for non-normally distributed data. The discriminatory performance of the TG/HDL-C ratio for identifying insulin resistance was examined by receiver operating characteristic (ROC) curve analysis, with the optimal threshold established according to the Youden index [18]. A p-value of less than 0.05 (two-tailed) was considered statistically significant.

The sample size was determined using G*Power version 3, (Heinrich-Heine-Universität Düsseldorf, Düsseldorf, Germany) to assess differences in the TG/HDL-C ratio between the participants with and without insulin resistance. Variability parameters were derived from previously published data [19]. Using a two-sided α level of 0.05 and 80% statistical power, the minimum required sample size was calculated as 140.

Ethics approval

This study was approved by the Derince Training and Research Hospital Clinical Research Ethics Committee (decision number: 2025-16).

## Results

Our study evaluated 207 children aged 5-18 years, with a median age of 12.4 years. Of these, 127 (61.3%) were female patients and 80 (38.7%) were male patients. Among the girls, 34 (26.8%) were prepubertal, and 93 (73.2%) were pubertal, whereas among the boys, 30 (37.5%) were prepubertal, and 50 (62.5%) were pubertal. According to sex- and puberty-specific HOMA-IR cutoffs, insulin resistance was identified in 92 (44.4%) patients, whereas 115 (55.6%) were insulin-sensitive. Acanthosis nigricans was detected in 70 (33.8%) patients, and elevated blood pressure was observed in 15 (7.2%). Table 1 presents the initial clinical and laboratory data. Compared to the group without insulin resistance, the group with insulin resistance showed significantly higher weight SDS (p=0.001), weight (p=0.009), BMI (p=0.001), and BMI SDS (p=0.028) values. Metabolic parameters, including fasting insulin, HOMA-IR, HbA1c, and fasting glucose, were significantly increased in the insulin resistance group (all p<0.001). Additionally, TG/HDL-C ratio (p=0.018), TG (p=0.007), and TSH levels (p=0.022) were higher in the group with insulin resistance. There were no significant differences in age, height, height SDS, ALT, AST, total cholesterol, HDL cholesterol, LDL cholesterol, cortisol, ACTH, or free T4 levels (all p>0.05) (Table 1).

**Table 1:** Baseline demographic, anthropometric, metabolic, and laboratory characteristics of children with and without insulin resistance Demographic, anthropometric, and biochemical characteristics of the participants according to insulin resistance status. Data are presented as median (interquartile range {IQR}). Insulin resistance was defined based on sex-specific and pubertal stage-specific HOMA-IR cutoff values *A statistically significant correlation (p<0.05) BMI, body mass index; IR, insulin resistance; SDS, standard deviation score; HDL, high-density lipoprotein; HOMA-IR, homeostasis model assessment for insulin resistance; HDL, high-density lipoprotein; TG/HDL, triglyceride to high-density lipoprotein; HbA1c, glycated hemoglobin; LDL, low-density lipoprotein; AST, aspartate aminotransferase; ACTH, adrenocorticotropic hormone; TSH, thyroid-stimulating hormone; ALT, alanine aminotransferase

Variables	IR negative (median, IQR)	IR positive (median, IQR)	P-value
Age (years)	12.2 (8.2-15.5)	12.8 (9.9-14.9)	0.141
Weight (kg)	70 (44-87)	79.6 (61.2-90.8)	0.009*
Weight SDS	2.7 (2.1-3.2)	3.1 (2.5-3.7)	0.001*
Height (cm)	154 (134-165)	157 (147-166)	0.071
Height SDS	0.45 ((-0.4)-1.4)	0.8 ((-0.1)-1.7)	0.082
BMI (kg/m²)	28.9 (25.4-31.3)	30.9 (27.3-34.3)	0.001*
BMI SDS	2.5 (2.2-2.9)	2.8 (2.3-3.1)	0.028*
HOMA-IR	2.3 (1.7-3.4)	5.2 (4-7)	<0.001*
TG/HDL	1.7 (1.2-2.8)	2.1 (1.6-3.1)	0.018*
Fasting insulin (µIU/mL)	10.1 (7.2-15.3)	22.3 (17.1-30.3)	<0.001*
Fasting glucose (mg/dL)	89 (86-95)	94 (89-101)	<0.001*
HbA1c (%)	5.2 (5-5.4)	5.4 (5.2-5.6)	<0.001*
Total cholesterol (mg/dL)	157 (138-172)	159.5 (144-177)	0.209
LDL cholesterol (mg/dL)	82.5 (72-99.3)	87.5 (75-104.8)	0.179
HDL cholesterol (mg/dL)	50.3 (44.1-58.7)	47.2 (42.2-55)	0.155
Triglycerides (mg/dL)	88 (64-128)	105.3 (77.5-137.75)	0.007*
ALT (U/L)	22 (16.8-28)	22.5 (18-32.8)	0.105
AST (U/L)	23 (19-27.3)	23 (19-29)	0.787
Cortisol (µg/dL)	8 (5.9-11)	8.7 (6.1-12.2)	0.718
ACTH (pg/mL)	15.8 (10.5-25.2)	20 (16.3-27)	0.24
TSH (µIU/mL)	2 (1.5-2.6)	2.2 (1.7-3.2)	0.022*
Free T4 (ng/dL)	1.02 (0.94-1.12)	1.0 (0.92-1.1)	0.415

HOMA-IR values were positively correlated with insulin, glucose, HbA1c, body mass index SDS, TG, and age and negatively correlated with HDL cholesterol. The TG/HDL-C ratio was positively correlated with insulin, glucose, ALT, AST, and HOMA-IR levels, although these relationships were weaker than those observed for HOMA-IR (Table 2).

**Table 2:** Correlations of HOMA-IR and triglyceride/HDL-C ratio with clinical, anthropometric, and biochemical parameters Spearman’s rank correlation was used to assess relationships between variables due to non-normal data distribution *A statistically significant correlation (p<0.05) BMI, body mass index; ALT, alanine aminotransferase; AST, aspartate aminotransferase; HOMA-IR, homeostasis model assessment for insulin resistance; LDL, low-density lipoprotein; TG, triglycerides; HbA1c, glycated hemoglobin; SDS, standard deviation score; HDL, high-density lipoprotein; ns, not significant

Variable	HOMA-IR	P-value	TG/HDL-C	P-value
Age (years)	0.415	<0.001	0.181	0.013*
Weight SDS	0.253	<0.001	-	ns
BMI SDS	0.21	0.002	-	ns
Glucose (mg/dL)	0.419	<0.001	0.181	0.013*
Insulin (µU/mL)	0.965	<0.001	0.295	<0.001*
HbA1c (%)	0.344	<0.001	-	ns
Triglycerides (mg/dL)	0.321	<0.001	0.943	<0.001*
HDL (mg/dL)	-0.216	0.002	-0.761	<0.001*
LDL (mg/dL)	-	ns	0.239	0.001*
Total cholesterol (mg/dL)	-	ns	0.229	0.002*
ALT (U/L)	0.165	0.02	0.255	0.001*
AST (U/L)	-	ns	0.229	0.002*

**Figure 1:**
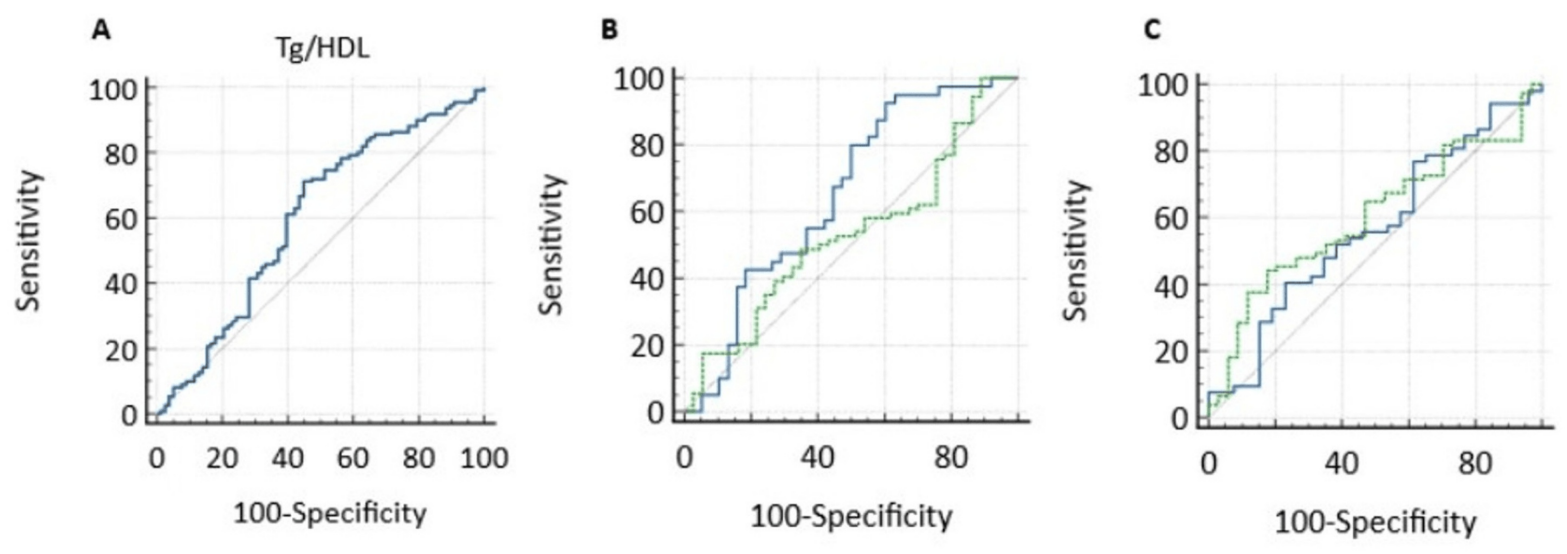
ROC curve analysis using the TG/HDL-C ratio to identify IR in the overall cohort (A), by sex (B), and by pubertal status (A) ROC analysis for TG/HDL-C (AUC=0.601; p=0.019). (B) Comparison of ROC analysis according to sex (green line, female; blue line, male) (p=0.157). (C) Comparison of ROC analysis according to puberty (green line, prepubertal; blue line, pubertal) (p=0.699) ROC, receiver operating characteristic; TG/HDL-C, triglyceride/high-density lipoprotein cholesterol; IR, insulin resistance; AUC, area under the curve

ROC analysis showed that the optimal TG/HDL-C cutoff value for predicting insulin resistance was approximately 1.7 (area under the curve {AUC}=0.601, p=0.019, 71.2% sensitivity, and 55.1% specificity) (Figure 1). ROC analysis was performed to evaluate the discriminative ability of the TG/HDL-C ratio in detecting insulin resistance at all stages of puberty. In the prepubertal group, the AUC was 0.566 (standard error {SE}=0.0699; 95% CI=0.449-0.678), and in the pubertal group, the AUC was 0.6 (SE=0.0558; 95% CI=0.503-0.692). The pairwise comparison of the ROC curves using the DeLong test showed that the difference between the AUCs was not statistically significant (difference=0.0346; 95% CI=-0.141-0.210; z=0.387; p=0.699). ROC analysis was performed to compare the diagnostic performance of the TG/HDL-C ratio in predicting insulin resistance according to sex. In the male group, the AUC was 0.653 (SE=0.0639; 95% CI=0.536-0.757), and in the female group, the AUC was 0.53 (SE=0.0581; 95% CI=0.433-0.626). The pairwise comparison of the ROC curves using the DeLong test showed that the difference in AUC between the sexes was not statistically significant (difference=0.122; 95% CI=-0.047-0.252; z=1.416; p=0.157).

## Discussion

Insulin suppresses lipolysis in adipocytes, thereby preventing the formation of free fatty acids from triglycerides (TG). In insulin resistance, as insulin’s effect on adipocytes decreases, the flow of free fatty acids from adipocytes to the liver significantly increases. This stimulates hepatic TG synthesis and the production of very low-density lipoprotein (VLDL), which is responsible for transporting TG in plasma. VLDL with a high TG content receives cholesterol esters from HDL via the enzyme cholesterol ester transfer protein (CETP), providing excess TG to HDL in return. As a result, HDL becomes rich in TG and poor in cholesterol levels. In insulin resistance, while lipoprotein lipase activity decreases, hepatic lipase activity increases. Decreased lipoprotein lipase reduces HDL formation from TG-rich lipoproteins, and increased hepatic lipase breaks down TG-rich HDL into smaller particles, accelerating the clearance of HDL particles. This process leads to an atherogenic lipid profile in plasma, characterized by hypertriglyceridemia and low HDL-C levels [20,21]. In addition, in insulin resistance, while insulin’s effect of inhibiting hepatic glucose production is reduced, its stimulatory effect on TG synthesis is partially preserved, which also contributes to the condition [22]. The rationale for employing the TG/HDL-C ratio as a measure of IR is based on this pathophysiological link between insulin resistance and dyslipidemia. In our study, as a clinical reflection of this pathophysiology, a positive association was found between TG/HDL-C and HOMA-IR.

Studies conducted in pediatric populations have reported different cutoff values for TG/HDL-C in predicting insulin resistance, reflecting the impact of ethnic, genetic, and methodological differences. While Giannini et al. [23] suggested a threshold of 2.27, Krawczyk et al. [24] reported a value of 3. In contrast, Behiry et al. defined a lower threshold of 1.36 and reported a sensitivity of 85.7% and specificity of 66.7% for this cutoff [12]. Similarly, Nur Zati Iwani et al. [25] proposed a cutoff value of 1.11, comparable to Behiry et al.’s [12] findings. In studies conducted in Turkey, Demiral found a positive association between TG/HDL-C and HOMA-IR but did not define a specific cutoff value [26]. Likewise, Ciftci and Kayas demonstrated that higher TG/HDL-C ratios were associated with increased fasting insulin and HOMA-IR levels [27]. Taken together, these studies demonstrate a consistent link between the TG/HDL-C ratio and insulin resistance; however, the threshold values identified in the literature vary considerably, spanning from approximately 1.3 to 3. In our study, a TG/HDL-C cutoff value of 1.7 yielded a sensitivity of 71.2% and specificity of 55.1%, with an AUC of 0.601, indicating modest discriminatory performance for identifying insulin resistance. These findings imply that this parameter may be useful as a standalone marker in settings where insulin measurement and HOMA-IR calculation are not feasible, such as primary healthcare; however, its moderate diagnostic accuracy should be taken into account. When insulin measurements are available, the TG/HDL-C ratio should be considered a supportive marker, and further studies are needed to determine whether it adds incremental value in multivariable predictive models. Ethnic differences in insulin sensitivity, lipid metabolism, body fat distribution, and lifestyle-related factors have been shown to influence both baseline lipid profiles and the relationship between dyslipidemia and insulin resistance in pediatric populations [23,28]. Therefore, the applicability of a single universal TG/HDL-C cutoff across different ethnic groups may be limited, supporting the need for population-specific validation studies.

In a large-scale study conducted among Korean adolescents, the TG/HDL-C ratio did not differ significantly between sexes [28]. Similarly, some studies have noted that the ability of the TG/HDL-C ratio to identify insulin resistance remains relatively stable regardless of age or pubertal stage [13]. In contrast, Liang et al. observed a marked increase in the TG/HDL-C ratio during adolescence and suggested higher cutoff values in girls than in boys [19]. In the present study, neither sex nor pubertal status significantly influenced the diagnostic performance of the TG/HDL-C ratio. When considered alongside existing literature, these findings highlight inconsistencies across populations, underscoring the likelihood that biological and environmental determinants of the TG/HDL-C ratio differ by ethnicity and region. Accordingly, population-specific evaluations of this ratio appear to be warranted.

In our study, children with insulin resistance exhibited more adverse metabolic profiles than their insulin-sensitive peers. Elevated TG concentrations, reduced HDL-C levels, and impaired glucose regulation were prominent features of this difference. These observations are consistent with previous reports emphasizing the close interplay between dyslipidemia, disrupted glucose metabolism, and insulin resistance in pediatric obesity [29]. Collectively, these findings reinforce the clinical importance of the early identification and ongoing monitoring of metabolic risk factors in children with insulin resistance.

The strengths of this study include the evaluation of the TG/HDL-C ratio, a biomarker for which data in children from our country remain limited; the establishment of a population-specific cutoff value; and the assessment of the influence of sex and pubertal status on diagnostic performance. Nevertheless, the retrospective, single-center design restricts generalizability and precludes causal inference.

## Conclusions

In the present study, we concluded that assessment based on the TG/HDL-C ratio demonstrated a moderate diagnostic performance in determining insulin resistance, that its diagnostic performance did not change significantly according to puberty status and gender, and that the threshold value of 1.7 could be used for children in our country. Considering these findings, it is thought that this parameter can be used alone in situations where insulin measurement and HOMA-IR calculation are not possible, such as in primary healthcare settings. However, in cases where insulin measurements are available, it should be considered a supportive marker for HOMA-IR.
